# Plin2-deficiency reduces lipophagy and results in increased lipid accumulation in the heart

**DOI:** 10.1038/s41598-019-43335-y

**Published:** 2019-05-06

**Authors:** Ismena Mardani, Knut Tomas Dalen, Christina Drevinge, Azra Miljanovic, Marcus Ståhlman, Martina Klevstig, Margareta Scharin Täng, Per Fogelstrand, Max Levin, Matias Ekstrand, Syam Nair, Björn Redfors, Elmir Omerovic, Linda Andersson, Alan R. Kimmel, Jan Borén, Malin C. Levin

**Affiliations:** 1 Department of Molecular and Clinical Medicine/Wallenberg Laboratory, Institute of Medicine, the Sahlgrenska Academy at University of Gothenburg and Sahlgrenska University Hospital, Gothenburg, Sweden; 2 Department of Nutrition, Institute of Basic Medical Sciences, University of Oslo, Oslo, Norway; 3 Centre of Perinatal Medicine and Health, Institute of Neuroscience and Physiology, The Sahlgrenska Academy at University of Gothenburg, Gothenburg, Sweden; 4 Laboratory of Cellular and Developmental Biology, National Institute of Diabetes and Digestive and Kidney Diseases, National Institutes of Health, Bethesda, MD USA

**Keywords:** Molecular biology, Molecular medicine

## Abstract

Myocardial dysfunction is commonly associated with accumulation of cardiac lipid droplets (LDs). Perilipin 2 (Plin2) is a LD protein that is involved in LD formation, stability and trafficking events within the cell. Even though Plin2 is highly expressed in the heart, little is known about its role in myocardial lipid storage. A recent report shows that cardiac overexpression of Plin2 result in massive myocardial steatosis suggesting that Plin2 stabilizes LDs. In this study, we hypothesized that deficiency in Plin2 would result in reduced myocardial lipid storage. In contrast to our hypothesis, we found increased accumulation of triglycerides in hearts, and specifically in cardiomyocytes, from *Plin2*^−/−^ mice. Although *Plin2*^−/−^ mice had markedly enhanced lipid levels in the heart, they had normal heart function under baseline conditions and under mild stress. However, after an induced myocardial infarction, stroke volume and cardiac output were reduced in *Plin2*^−/−^ mice compared with *Plin2*^+/+^ mice. We further demonstrated that the increased triglyceride accumulation in Plin2-deficient hearts was caused by altered lipophagy. Together, our data show that Plin2 is important for proper hydrolysis of LDs.

## Introduction

The mammalian heart has a high energy demand in order to sustain its contractile function. Although circulating lipids are the major energy source, myocardial triglycerides constitute an important storage pool of energy for the heart^1,2^. An accessible triglyceride reserve is essential to meet alterations in energy demand and to compensate for the fluctuating availability of fatty acids (FAs) in the plasma^3^. Lipid homeostasis in cardiomyocytes relies on a critical balance between fatty acid uptake from the surrounding and consumption by mitochondrial β-oxidation. Myocardial triglyceride storage is generally low under normal conditions, but lipid storage is increased with fasting or high-fat feeding^4,5^ and in pathological conditions, such as myocardial ischemia^6–8^.

Triglycerides are stored intracellularly in cytosolic lipid droplets (LDs)^9,10^, and FAs are mobilized from the triglyceride reserve in LDs when energy demand increases. Traditionally, lipolysis by LD-associated lipases has been believed to be the main pathway for mobilization of FAs. Recently, it has also been recognized that lipids can be mobilized from LDs by lipophagy^11^. During this process, LDs are delivered to lysosomes where they are hydrolyzed by lysosomal lipases^12,13^. Lipophagy of LDs during conditions of cell starvation seems to be required to preserve mitochondrial respiration^14^.

LDs consist of a core of neutral lipids enclosed in a phospholipid monolayer with numerous proteins of importance for LD hydrolysis^9,10^.The most abundant LD proteins are perilipins (Plins), which play an important role in the regulation of lipid storage, for example by shielding lipid droplets from lipase activity^15^. There are 5 separate Plins (Plin1-5), and they differ in their tissue distribution and function^15,16^. Plin1 and Plin4 are mostly expressed in adipose tissue, and Plin2 and Plin3 are widely expressed in the the body. Plin5 is mainly expressed in oxidative tissues, such as heart and is the most well-studied perilipin in this tissue^15,16^. We and others have previously shown that deficiency in Plin5 in mice results in a dramatic reduction in triglyceride accumulation in the heart^6,17^. Furthermore, although Plin5 deficiency does not affect heart function under normal conditions, heart function is reduced during stress or myocardial ischemia in *Plin5*^−/−^ mice^6^, indicating the importance of Plin5 in myocardial lipid dynamics and function.

Even though Plin2 is highly expressed in the heart, little is known about its role in myocardial lipid storage. A recent report shows that cardiac overexpression of Plin2 resulted in massive myocardial steatosis^18^, suggesting that Plin2 stabilizes LDs. Studies focusing on hepatocytes show that *Plin2*^−/−^ mice have reduced lipid accumulation in the liver and are protected against diet-induced liver steatosis^15,19–21^. Furthermore, *in vitro* studies suggest that deficiency in Plin2 reduces lipid accumulation through loss of lipase barrier protection in LDs, resulting in increased lipolysis^15,22^. Importantly, recent research suggest that Plin2 is also involved in targeting LDs for lipophagy^23,24^. Consequently, the exact role of Plin2 in regards to LD hydrolysis, in tissues in general and in cardiomyocytes specifically, remains to be clarified.

In this study, we hypothesized that deficiency in Plin2 would result in reduced myocardial lipid storage and improved cardiac function following myocardial ischemia. Contrary to our expectations, we found that Plin2-deficiency resulted in increased myocardial lipid storage and reduced cardiac function following a myocardial infarction. We used Plin2-deficient mice and isolated primary cardiomyocytes to demonstrate that the increased triglyceride accumulation in Plin2-deficient hearts was caused by reduced lipophagy.

## Results

### Plin2 protein associates with triglyceride levels in cardiomyocytes

**Figure 1:**
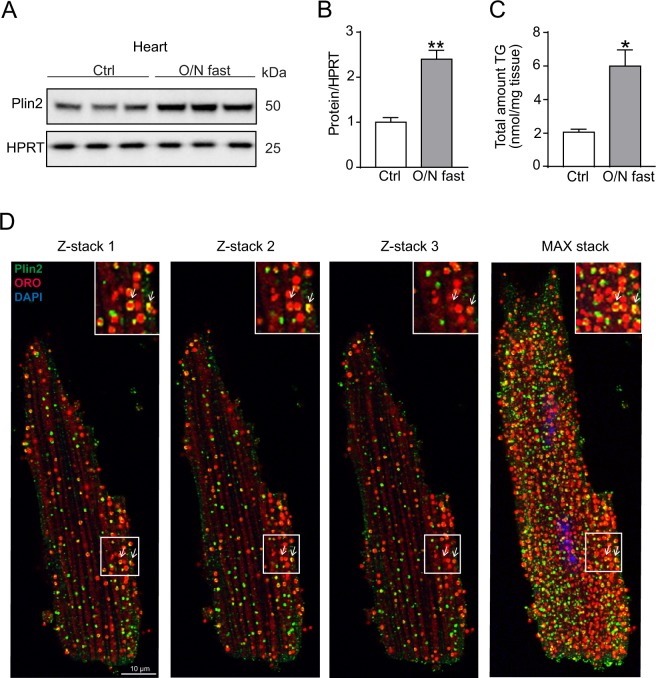
Regulation of Plin2. (**A**) Immunoblot analysis of Plin2 using protein lysates from control (refed) and O/N fasted C57Bl/6N mice hearts (n = 3). Full-length blots are presented in Supplementary Fig. S7. (**B**) Quantification of immunoblot analysis on Plin2 (n = 3). (**C**) Triglyceride content in hearts of control (refed) and overnight fasted mice (n = 3). (**D**) Confocal microscopy images on C57Bl/6N cardiomyocytes, shown as z-stack 1, 2, 3 and a max stack picture containing 30 z-stacks compromised into one image. Plin2 is shown in green, ORO (LDs) in red and nuclei in blue and co-localization of LDs and Plin2 in yellow (x630 magnification, scale bar: 10 µm). Data are presented as mean ± SEM, *p < 0.05 *vs. Plin2*^+/+^, **p < 0.01 *vs. Plin2*^+/+^. TG, triglycerides; O/N, overnight.

Plin2 has previously been shown to be induced by fatty acids in cell culture^25^ and here we tested whether Plin2 protein levels associate with lipid accumulation in the heart. To induce lipid accumulation, we fasted *Plin2*^+/+^ mice overnight and found marked increases in Plin2 protein levels in parallel with increased triglyceride levels in hearts from the fasted mice compared to hearts from control (refed) mice (Fig. 1A–C). Using immunohistochemistry, we also showed that Plin2 localized to LDs within isolated primary cardiomyocytes (Fig. 1D). These results indicate that Plin2 protein levels associate with increased lipid storage and that Plin2 coats myocardial LDs.

### Plin2 deficiency promotes triglyceride accumulation in cardiomyocytes

**Figure 2:**
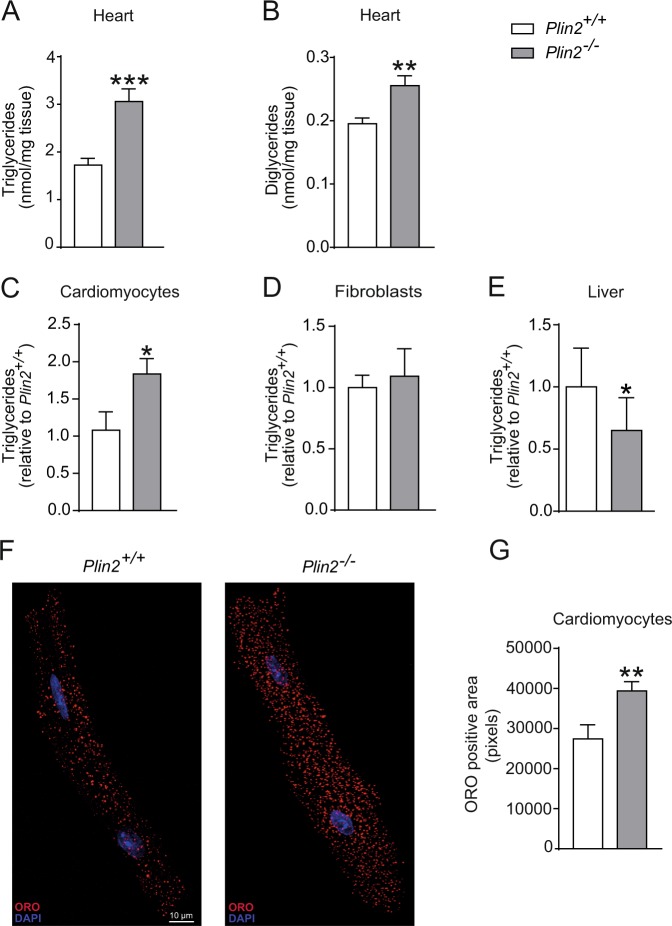
Increased myocardial triglyceride accumulation in *Plin2*^−/−^ hearts. (**A**) Triglyceride and (**B**) diglyceride content in hearts of 8-week-old *Plin2*^+/+^ and *Plin2*^−/−^ mice after 4 hours fasting (n = 6–7). (**C**) Triglyceride content in cardiomyocytes isolated from *Plin2*^+/+^ and *Plin2*^−/−^ hearts after O/N fast (n = 5–7). (**D**) Triglyceride content in fibroblasts isolated from *Plin2*^+/+^ and *Plin2*^−/−^ hearts after O/N fast (n = 4). (**E**) Triglyceride content in liver of *Plin2*^+/+^ and *Plin2*^−/−^ mice after 4 hours fasting (n = 4–5). (**F**) Confocal microscopy images on *Plin2*^+/+^ and *Plin2*^−/−^ cardiomyocytes, shown as a merged picture containing 10 z-stacks compressed into one image. LDs are shown in red and nuclei in blue (x630 magnification, scale bar: 10 µm). (**G**) Quantification of ORO positive area (pixels) in *Plin2*^+/+^ and *Plin2*^−/−^ cardiomyocytes (n = 4–5). Data are presented as mean ± SEM, *p < 0.05 *vs. Plin2*^+/+^, **p < 0.01 *vs. Plin2*^+/+^ and ***p < 0.001 *vs*. *Plin2*^+/+^; TG, triglycerides; DG, diglyceride; O/N, overnight.

Because cardiomyocyte-specific overexpression of Plin2 occurs with cardiac steatosis^18^, we tested if deficiency in Plin2 resulted in reduced myocardial lipid storage using *Plin2*^−/−^ mice. We first confirmed that mRNA and protein expression of Plin2 was absent in the hearts of *Plin2*^−/−^ mice (Fig. S1A,B). Body weight and circulating levels of lipids, glucose and insulin did not differ between *Plin2*^+/+^ and *Plin2*^−/−^ mice on a chow diet (Table S1). We performed lipid analysis of heart tissue from *Plin2*^+/+^ and *Plin2*^−/−^ mice, and surprisingly found that levels of triglycerides and diglycerides were increased in *Plin2*^−/−^ hearts compared to *Plin2*^+/+^ hearts (Fig. 2A,B). Other analysed lipids were not altered (Fig. S2). Furthermore, we investigated whether the increased accumulation of triglycerides seen in the hearts of *Plin2*^−/−^ mice could be cardiomyocyte specific. In line with this hypothesis, we observed increased levels of triglycerides in isolated primary cardiomyocytes (Fig. 2C**)** but not in cardiac fibroblasts (Fig. 2D) from *Plin2*^−/−^ versus Plin2^+/+^ mice. In agreement, we also observed increased ORO-stained LDs in cardiomyocytes isolated from *Plin2*^−/−^ versus *Plin2*^+/+^ mice (Fig. 2F,G). In contrast to our observation of increased triglyceride accumulation in hearts and cardiomyocytes from *Plin2*^−/−^ mice, we showed that triglyceride levels were reduced in liver from *Plin2*^−/−^ compared with *Plin2*^+/+^ mice (Fig. 2E), consistent with earlier studies^15,19^.

### Heart function is compromised by pathological stress in *Plin2*^−/−^ mice

**Figure 3:**
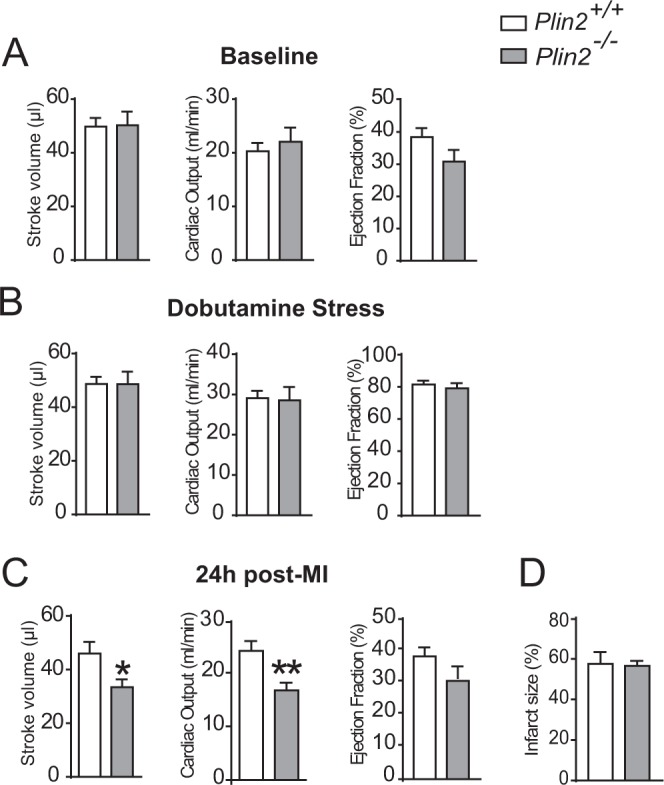
Reduced heart function following myocardial infarction in *Plin2*^−/−^ mice. Heart function assessed with echocardiography, showing stroke volume, cardiac output and ejection fraction from *Plin2*^+/+^ and *Plin2*^−/−^ mice in baseline condition (**A**), following dobutamine stress (**B**) and 24 h following myocardial infarction (**C**) (n = 8–9). Infarct size in *Plin2*^+/+^ and *Plin2*^−/−^ mice 24 h following a myocardial infarction (**D**). Data presented as mean ± SEM, *p < 0.05 *vs. Plin2*^+/+^, **p < 0.01 *vs. Plin2*^+/+^

Because there is a correlation between myocardial lipid accumulation and heart function^26^, we investigated whether heart function was affected by Plin2 deficiency. We did not observe any differences in heart function or dimensions between Plin2^+/+^ and *Plin2*^−/−^ mice under baseline conditions or after dobutamine-induced stress (Table 1 and Fig. S3), suggesting that *Plin2*^−/−^ mice maintain a normal heart function even though triglyceride levels in cardiomyocytes are markedly increased. In addition, expression of natriuretic peptides was not significantly affected (Fig. S3A) and there was no difference in cardiomyocyte size (Fig. S3B–D). Next, we wanted to assess how Plin2-deficient hearts performed under pathological stress and therefore induced a myocardial infarction (induced by ligating the left anterior descending coronary artery). Importantly, we observed a reduction in stroke volume and cardiac output in *Plin2*^−/−^ compared with *Plin2*^+/+^ mice 24 hours after induced MI (Fig. 3). Thus, our data indicate that *Plin2*^−/−^ mice have a normal heart function under baseline conditions and under mild stress, but they perform worse following pathological stress.

**Table 1:** Baseline and dobutamine stress echocardiographic analysis.

	Baseline		p	Dobutamine stress		p
	Plin2^+/+^	Plin2^−/−^		Plin2^+/+^	Plin2^−/−^	
n	9	8	ns	9	7	ns
LV V(d) (µL)	86.3 ± 11.4	89.9 ± 15.5	ns	59.6 ± 7.93	63.92 ± 8.89	ns
LV V(s) (µL)	36.6 ± 5.8	39.7 ± 6.3	ns	11.02 ± 4.61	11.89 ± 4.61	ns
EF (%)	57.3 ± 6.3	55.1 ± 7.4	ns	81.4 ± 7.30	81.09 ± 7.62	ns
SV (µL)	49.7 ± 9.6	50.2 ± 14.4	ns	48.6 ± 8.09	52.03 ± 9.64	ns
CO (ml/min)	20.2 ± 4.6	22.1 ± 7.4	ns	29.1 ± 5.41	31.18 ± 6.32	ns
CI (µl/g)	0.57 ± 0.09	0.60 ± 0.13	ns	0.80 ± 0.11	0.87 ± 0.22	ns

LV V(d), left ventricular diastolic volume; LV V(s), left ventricular systolic volume; EF, ejection fraction; SV, stroke volume; CO, cardiac output; CI, cardiac index. Data are presented as mean ± SD.

### Plin2-deficient cardiomyocytes have normal mitochondrial respiration

**Figure 4:**
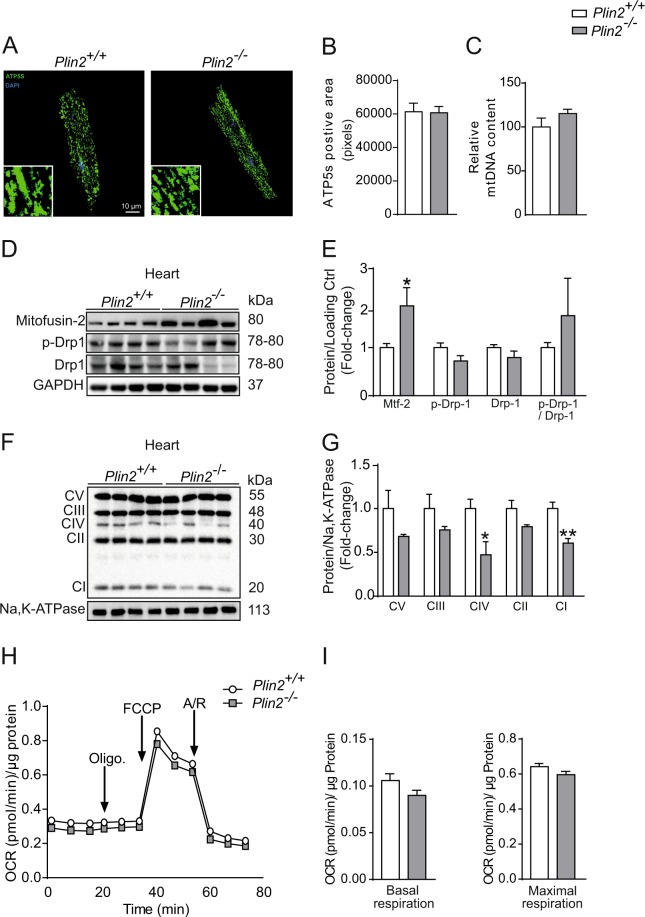
Plin2-deficient cardiomyocytes have normal mitochondrial respiration (**A**) Representative confocal microscopy images of cardiomyocytes isolated from *Plin2*^+/+^ and *Plin2*^−/−^ mice, shown as a max-stack containing 20 z-stack pictures compressed into one image, and insets show images with higher magnification. ATP5S is shown in green and nuclei staining in blue (x630 magnification, scale bar: 10 µm). (**B**) Quantification of ATP5s positive area (pixels) in *Plin2*^+/+^ and *Plin2*^−/−^ cardiomyoctyes (n = 4–5). (**C**) Mitochondrial DNA (mtDNA) content (normalized to the nuclear single-copy gene β-actin) in primary cardiomyocytes isolated from *Plin2*^+/+^ and *Plin2*^−/−^ mice (n = 3). (**D**) Representative immunoblot analysis of Mitofusin-2, p-Drp-1 and Drp-1, using heart homogenates from *Plin2*^+/+^ and *Plin2*^−/−^ hearts. GAPDH and HPRT was used as loading control. Full-length blots are presented in Supplementary Fig. S8. (**E**) Quantification shown as fold-change compared to *Plin2*^+/+^ (n = 8–9). (**F**) Immunoblot analysis of OXPHOS proteins. Full-length blots are presented in Supplementary Fig. S8. (**G**) Quantification of F (n = 4). (**H**) Seahorse analysis of oxygen consumption rates (OCR) in primary cardiomyocytes isolated from *Plin2*^+/+^ and *Plin2*^−/−^ mice. OCR were assessed under basal condition and following the addition of 1 µM Oligomyocin, 0.25 µM FCCP and 2 µM Antimycin A and Rotenone (n = 6–7 isolations of cardiomyocytes per genotype; each experiment performed in 12 wells). (**I**) The rates of basal respiration and maximal respiration are calculated as described in the method section. OCR was normalized to total cellular protein content for each well. Data are presented as mean ± SEM, *p < 0.05 *vs. Plin2*^+/+^ and **p < 0.01 *vs. Plin2*^+/+^.

Next, we aimed to clarify why Plin2-deficiency results in increased myocardial lipid storage. Because catabolism of lipids in cardiomyocytes depend mainly on mitochondrial function, we assessed whether Plin2-deficient cardiomyocytes had a reduced mitochondrial function. First, we evaluated the mitochondrial ultrastructure by staining isolated cardiomyocytes for the mitochondrial marker ATP synthase. There were no major differences between mitochondrial density or ultrastructure in *Plin2*^+/+^ and *Plin2*^−/−^ cardiomyocytes (Fig. 4A–C). Western blots showed that the fusion marker mitofusin2 was significantly upregulated in *Plin2*^−/−^ hearts, but the fission marker Drp1 was not affected (Fig. 4D,E). Mitochondrial protein expression of OXPHOS proteins CIV and CI (Fig. 4F,G) was reduced in *Plin2*^−/−^ hearts, suggesting that mitochondrial respiration may be affected in *Plin2*^−/−^ hearts. To clarify if there is a difference in mitochondrial respiration, we meassured oxygen consumption rates (OCR) in isolated cardiomyocytes using the Seahorse metabolic assay. Basal and maximal mitochondrial respiration did not differ between *Plin2*^+/+^ and *Plin2*^−/−^ cardiomyocytes (Fig. 4H,I). We further elucidated whether this was accurate specifically for fatty acid oxidation and we could not see any differences in produced CO_2_ or ASM between *Plin2*^+/+^ and *Plin2*^−/−^ hearts (Fig. S4). Thus, our results show that mitochondrial function is intact in *Plin2*^−/−^ cardiomyocytes and that the increased triglyceride accumulation in *Plin2*^−/−^ cardiomyocytes is not due to differences in respiration.

### Reduced lipophagy in *Plin2*^−/−^ cardiomyocytes

**Figure 5:**
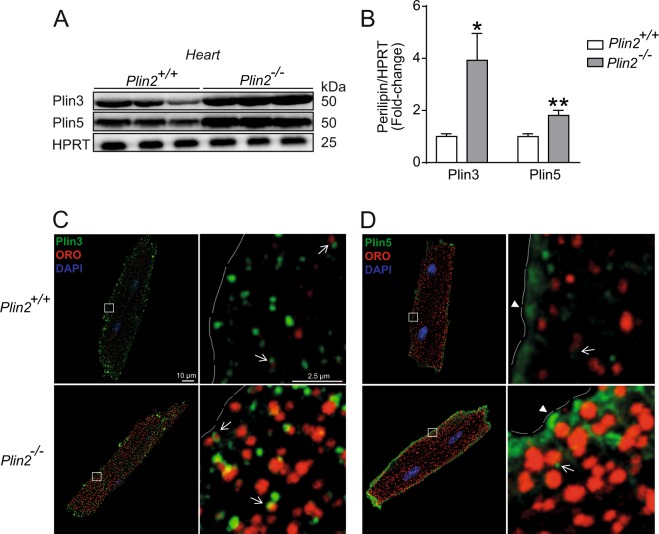
Elevated Plin3 and Plin5 protein expression in *Plin2*^−/−^ hearts. (**A**) Representative figure of immunoblot analysis of Plin3 and Plin5 using protein lysates from *Plin2*^+/+^ and *Plin2*^−/−^ hearts. Full-length blots are presented in Supplementary Fig. S9. (**B**) Quantification of immunoblot analysis on Plin3 and Plin5 (n = 8–9). Data are presented as mean ± SEM, *p < 0.05 *vs. Plin2*^+/+^ and **p < 0.01 *vs. Plin2*^+/+^. (**C**,**D**) Representative confocal microscopy images of *Plin2*^+/+^ and *Plin2*^−/−^ cardiomyocytes. Images are shown as a max stack containing 10 z-stacks compressed into one single image and a higher magnification z-stack image. (**C**) Plin3 is shown in green, ORO (LDs) in red, nuclei in blue and Plin3 and lipid droplet co-localization in yellow. (**D**) Plin5 is shown in green, LDs in red, nuclei in blue. Co-localization of Plin5 and LD is indicated with arrows, whereas Plin5-positive staining without association to LDs are shown with arrow heads (x630 magnification, scale bar 10 µm, higher magnification image scale bar 2, 5 µm).

Subsequently, we determined whether the increased myocardial lipid storage in Plin2 deficient hearts could be explained by increased lipid uptake in cardiomyocytes. However, we found that fatty acid uptake was not affected in *Plin2*^−/−^ hearts, nor was the expression of key regulators of myocardial lipid uptake (Fig. S5). Next, we assessed if Plin2-deficiency affected proteins that reduce mobilization of triglycerides from LDs, resulting in increased LD accumulation. First, we measured whether Plin2 deficiency was accompanied by modified levels of other perilipins in the heart. There were no differences in mRNA expression of Plin3, Plin4 and Plin5 in hearts from *Plin2*^−/−^ versus *Plin2*^+/+^ mice (Fig. S6A), but importantly Plin3 and Plin5 protein levels were higher in *Plin2*^−/−^ hearts (Fig. 5A,B). Plin3 and Plin5 have previously been described as exchangeable LD proteins, not only existing on the LD surface but also in the cytosol^16^. We therefore tested if the increased Plin3 and Plin5 abundance in *Plin2*^−/−^ hearts was confined specifically to LDs. We showed that Plin3 was mainly located on evenly dispersed LDs throughout the cardiomyocyte, with a similar pattern in *Plin2*^+/+^ and *Plin2*^−/−^ cardiomyocytes (Fig. 5C). Plin5 also co-localized with LDs to some extent. However, a large amount of Plin5-positive staining was not positive for ORO, but localized to larger vesicle structures near the plasma membrane of the cardiomyocytes. These Plin5-positive structures were even more distinct in *Plin2*^−/−^ compared with *Plin2*^+/+^ cardiomyocytes (Fig. 5D).

**Figure 6:**
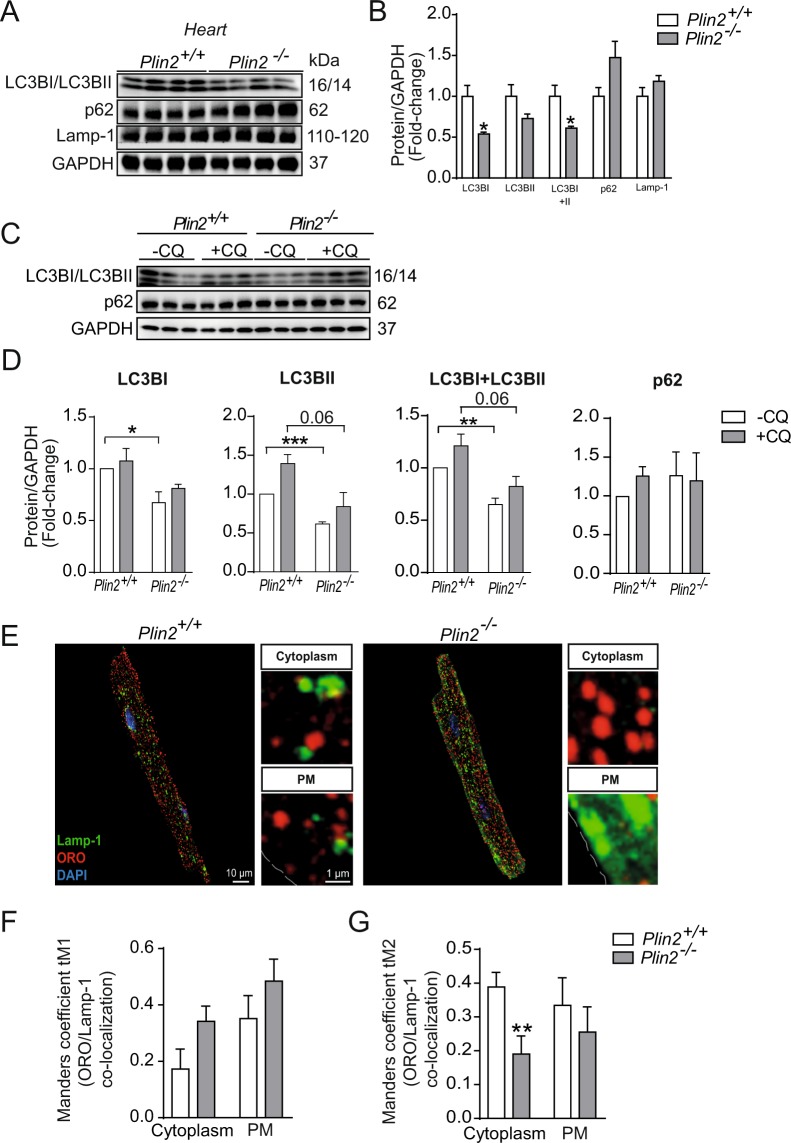
Reduced lipophagy in Plin2^−/−^ hearts. (**A**) Immunblot analysis of LC3BI, LC3BII, p62 and Lamp-1 in lysates from *Plin2*^+/+^ and *Plin2*^−/−^ hearts after O/N fasting. Full-length blots are presented in Supplementary Fig. S10. (**B**) Quantification of immunoblot analysis in A (n = 4). (**C**) Immunblot analysis of LC3BI, LC3BII and p62 in cardiomyocytes, treated with or without 25 µM chloroquine, isolated from *Plin2*^+/+^ and *Plin2*^−/−^ mice. Full-length blots are presented in Supplementary Fig. S11. (**D**) Quantification of immunoblot analysis in C (n = 4). (**E**) Representative confocal microscopy images of *Plin2*^+/+^ and *Plin2*^−/−^ cardiomyocytes, shown as a max stack containing 10 z-stack images compressed into one single image, and a higher magnification z-stack image. Lamp-1 (lysosomes) is shown in green and ORO (LDs) is shown in red, nuclei in blue, and co-localization of Lamp-1 and ORO as as yellow (x630 magnification, scale bar 10 µm, inset image scale bar 1 µm). (**F**,**G**) Quantification of co-localization between lysosomes and lipid droplets. (**F**) Quantification of Manders coefficient tM1 (fraction of ORO in co-localization with Lamp-1) in cytosol and plasma membrane, and (**G**) Manders coefficient tM2 (fraction of Lamp-1 in co-localization with ORO) in cytosol and plasma membrane in cardiomyocytes isolated from *Plin2*^+/+^ and *Plin2*^−/−^ mice, (n = 4–5). Data are presented as mean ± SEM, *p < 0.05 *vs. Plin2*^+/+^.

Because LDs can also be hydrolyzed through lipophagy in lysosomes, we investigated if Plin2-deficiency affected the status of the autophagy/lysosomal system. Importanly, immunoblots from heart lysates showed reduced levels of the autophagy-marker LC3BI and II but similar levels of p62 **(**Fig. 6A,B**)**, suggesting reduced rates of autophagy. Next, we assessed autophagy/lipophagy markers in primary cardiomyocytes isolated from *Plin2*^+/+^ and *Plin2*^−/−^ hearts. In this pure population of cardiomyocytes, there was an evident reduction in LC3BI, LC3BII and total LC3B levels in *Plin2*^−/−^ cardiomyocytes (Fig. 6C,D). To evaluate autophagic flux, we blocked autophagosome breakdown with chloroquine treatment and found a trend in reduced LC3BII and total LC3B levels in the *Plin2*^−/−^ cardiomyocytes (Fig. 6C,D), indicating that the differences in LC3BII is not due to an increased autophagic flux but instead reduced autophagy/lipophagy. Moreover, protein levels of the lysosome marker Lamp1 was not affected (Fig. 6A,B), but importantly the lysosome localization is markedly affected in *Plin2*^−/−^ cardiomyocytes (Fig. 6E–G). Absence of Plin2 decreased co-localization of LDs with lysosomes (Fig. 6E–G), consistent with reduced autophagy/lipophagy. Furthermore, levels of phosphorylated AMPK were markedly reduced in *Plin2*^−/−^ hearts, indicating that pathways regulating lipophagy are suppressed (Fig. S6B,C). Collectively, our findings showing that Plin2-deficiency results in reduced lipophagy of LDs.

## Discussion

In this study, we investigated the role of Plin2 in myocardial lipid storage. We showed that Plin2 protein levels increased in parallel with lipid accumulation and that Plin2 is located to LDs in cardiomyocytes. Contrary to our expectations, we found that *Plin2*^−/−^ mice had increased myocardial triglyceride levels and increased myocardial abundance of Plin3 and Plin5 compared with *Plin2*^+/+^ mice. However, this increase in triglyceride levels was not seen in cardiac fibroblasts or in liver, suggesting a cardiomyocyte-specific increase in triglyceride storage in *Plin2*^−/−^ mice. We demonstrated that the increased triglyceride accumulation in Plin2-deficient hearts was caused by reduced lipophagy. Thus, our results suggest that Plin2 is important for proper hydrolysis of LDs.

Our findings demonstrate that Plin2 deficiency results in increased myocardial accumulation of triglycerides, seen in heart as well as in isolated cardiomyocytes. In recent years, lipid mobilization from LDs has been increasingly linked to lipophagy (i.e. autophagic digestion of LDs in lysosomes)^11,27^. Here, we demonstrate that the increased triglyceride accumulation found in *Plin2*^−/−^ hearts is the result of reduced lipophagy. The autophagy marker LC3B is lowered and lysosomes display reduced co-localization with LDs in *Plin2*^−/−^ cardiomyocytes. Lipophagy, together with lipolysis, plays a critical role for energy metabolism during fasting. The molecular mechanisms regulating the interplay between lipolysis and lipophagy is not well investigated, but may involve mammalian target of rapamycin (mTOR) and/or AMP-activated protein kinase (AMPK).

Recently, Plin2 has been identified as a target for chaperone-mediated autophagy (CMA), where removal of Plin2 by autophagy enable lipases (such as ATGL) to access the LD surface and thereby increase lipolysis^23^. Consequently, Plin2-deficiency would potentially result in free access for lipases to the LD surface, high rates of lipolysis and low levels of accumulating LDs. However, we find increased levels of accumulating triglycerides in *Plin2*^−/−^ cardiomyocytes. Thus, our results suggest that Plin2 not only may be involved in CMA and facilitating lipolysis, but that Plin2 are crucial for regulation of lipophagy. However, the mechanism of how Plin2 deficiency is linked to lipophagy and lysosomal activities needs to be further elucidated. Potentially, Plin2 may be important for “priming” LDs for lipophagy.

Importantly, our results showed that Plin2-deficiency reduced lipophagy in the heart. Plin2 deficiency resulted in increased protein abundance of Plin3 and Plin5 in the heart. Whether this is compensation for the absence of Plin2 or a result from the increased LD accumulation is still unknown. Nevertheless, our study suggests that there is an important interplay between perilipins, which controls accessibility of the triglyceride pool and determines hydrolysis of LDs. Plin3 clearly co-localized to LDs in cardiomyocytes and Plin3-coated LDs are evenly distributed within the cardiomyocytes. Plin5 also localizes to LDs, but not to the same extent as Plin3. Surprisingly, a large amount of Plin5 is expressed in the sub-sarcolemma of the cardiomyocytes, as shown by z-stacks of longitudinal confocal sections of cardiomyocytes.

In contrast to our results in heart, triglyceride accumulation was reduced in *Plin2*^−/−^ livers, consistent with previous reports^15,19^. There are serveral possible reasons for these functional differences. For example, specialized cell types may differentially utilize lipolysis versus lipophagy for their starvation and the increased levels of Plin5 in *Plin2*^−/−^ cardiomyocytes may have stabilizing functions for lipids. The autophagic processes (including lipophagy) have proven to be extremely important processes in the heart^28,29^.

Myocardial ischemia is associated with dysfunctional metabolism and accumulation of lipids. In this study, we found that deficiency in Plin2 resulted in reduced heart function following myocardial infarction. We have previously shown that *Plin5*^−/−^ mice have reduced heart function and outcome after myocardial ischemia^6^. This is intriguing due to the opposite effect of Plin2 and Plin5 ablation on myocardial lipid content. Indeed, Plin5 is the only perilipin known to mediate the association between lipid droplets and mitochondria^30^. Thus, the *Plin5*^−/−^ mice hearts have a diminished contact between mitochondria and lipid droplets combined with a reduced storage pool of lipids, resulting in a reduced heart function after myocardial infarction. Conversely, *Plin2*^−/−^ mice have increased triglyceride content and an altered composition of perilipins coating the lipid droplets, with an absence of Plin2 and increased concentrations of Plin3 and Plin5. It remains to be elucidated how this affects the protection against lipolysis and subsequent lipid utilization. However, studies in human skeletal muscle suggest a preferential utilization of Plin2 coated lipid droplets during moderate-intensity exercise^31^. Hence, the absence of Plin2 in hearts with a higher workload due to a myocardial infarction may affect the substrate availability and utilization and thereby reduce the heart function.

In conclusion, our study suggests that Plin2 regulates cellular lipid metabolism in a tissue-specific fashion. Furthermore, our results suggest that the composition of proteins coating the LDs, not only the amount of droplets, is important for myocardial function.

## Methods

### Mice

C57Bl/6N male mice (Taconic), *Plin2*^−/−^ mice^22^ and *Plin2*^+/+^ littermates (backcrossed onto C57Bl/6N for 10 generations) were housed in a pathogen-free barrier facility and fed with rodent chow diet (consisting of 12% calories from protein, 12% from fat and 66% from carbohydrates). *Plin2*^*+/–*^ heterozygous breeding was used, and *Plin2*^+/+^ and *Plin2*^−/−^ littermates were used for all experiments. All animal studies were approved by the Gothenburg animal ethics committee and conform to the guidelines from Directive 2010/63/EU of the European Parliament on the protection of animals used for scientific purposes. At the end of experiments, mice were sacrificed using isoflurane and cervical dislocation.

### mRNA Expression in Heart Tissue from Mice

Total RNA was extracted from snap-frozen mouse heart tissue using RNeasy Fibrous Tissue Kit (QIAGEN). cDNA was synthesized using the high-capacity cDNA Reverse Transcription Kit (Applied Biosystems) with random primers. mRNA expression of genes of interest was analysed with TaqMan real-time PCR in an ABI Prism 7900 HT Detection System (Applied Biosystems). The following TaqMan Gene Expression assays were used: Plin2 Mm00558672_m1; Plin3 Mm04208646_g1; Plin4 Mm00491061_m1; Plin5 Mm00508852_m1; and HPRT Mm01545399_m1 as an internal control.

### Western Blot Analysis

Frozen heart tissue was homogenized and the proteins were extracted with the Qproteosome Mammalian Protein Prep kit (Qiagen). Cells were lysed with Cell Lysis Buffer (10×) (Cell signaling) supplemented with PMSF. The samples were sonicated and centrifuged at 10 000 g for 10 minutes. The supernatant was collected and protein concentration was determined with the BCA protein assay kit (Pierce, ThermoScience). Equal amounts of total protein were loaded and separated on a NuPAGE 4–12% Bis-Tris gel or NuPAGE 4–20% Tris-Glycine gel (Invitrogen) and transferred to a nitrocellulose membrane (GE healthcare) or PVDF membrane (Bio-rad). Blots were probed with antibodies for Plin2 (20R-AP002; Fitzgerald), Plin3 (GP37; Progen), Plin5 (GP31; Progen), Mitofusin-2 (ab124773; abcam), Drp-1 (8570; Cell signaling), p-Drp-1 (4867; Cell signaling) UCP3 (ab3477; abcam), OXPHOS (MS604; abcam), p62 (5114 S; Cell signaling), LC3B (2775S; Cell signaling), p-AMPK (t172; Cell signaling), AMPK (23A3; Cell signaling), GAPDH (ab8245; abcam), Na,K ATPase (05–369; Millipore) and HPRT (ab109021: Abcam) and then with the corresponding horseradish peroxidase–conjugated secondary antibody. Immunoblots were visualized with Immobilon Western Chemiluminiscent horseradish peroxidase substrate (Millipore) and visualized with a digital camera (Biorad). Bands were quantified with Image lab software (Bio-rad) and normalized to either GAPDH, HPRT or Na,K ATPase.

### Isolation of primary cardiomyocytes and cardiac fibroblasts

Mice were anesthetized with isoflurane and the heart was rapidly excised, the aorta was cannulated and the heart was perfused retrogradely, initially with perfusion buffer alone (120 mM NaCl, 14.7 mM KCl, 0.6 mM KH_2_PO_4_, 0.6 mM Na_2_HPO_4,_ 1.2 mM MgSO_4_, 10 mM Na-Hepes, 5.5 mM Glucose, 4.6 mM NaHCO_3_, 30 mM Taurine, 10 mM BDM, pH 7) for 4 minutes, and the with the addition of Collagenase type 2 (230 u/mg dw) (Worthington) for 3 minutes and thereafter with digestion buffer together with 100 mM CaCl_2_ for 8 minutes on a Langendorff system (PanLab). After collagenase inhibition with fetal calf serum (FCS) (Thermofisher) the heart was torn apart and cardiomyocytes were separated from non-cardiomyocytes by centrifugation at 20 g for 3 minutes, the supernatant containing non-cardiomyocytes was used for fibroblast isolation. Ca^2+^ was reintroduced to the cardiomyocytes stepwise with increasing calcium concentrations until a final concentration of 900 mg/ml was achieved. After the calcium re-introduction, the cells were seeded in plating medium (MEM w Hanks BSS (Lonza), 10% Calf Serum, 10 mM BDM (Sigma Aldrich), 100 U/ml pencillin (Hyclone), 2 mM L-glutamine (Hyclone)) that had been calibrated in 37 °C, 18% O_2_ and 2% CO_2_ to reach an optimal pH, on plates coated with laminin (10 µg/well) (Thermofisher Scientific). The cardiac fibroblasts were isolated from the non-cardiomyocyte containg supernatant by centrifugation for 5 minutes at 2000 rpm and resuspended in fibroblasts medium (MEM w Hanks BSS, 10% Calf serum, 100 U/ml Pencillin, 2 mM L-glutamine). After a second centrifugation cells were seeded and incubated in 37 °C, 18% O_2_ and 5% CO_2_ according to previous publication^32^.

For lipid analysis and immunoflourescent stainings, the cardiomyocytes were harvested after 2 hours of incubation in 37 °C, 18% O_2_ and 2% CO_2_.

### Lipid Analysis

Tissue from 8-week-old *Plin2*^+/+^ and *Plin2*^−/−^ mice was excised and quickly washed in PBS and thereafter snap frozen in nitrogen. 50–100 mg tissue was homogenized in methanol using a combination of Precellys 24 homogenizer (Bertin Technologies) and Mixer Mill equipment (Retsch). Lipids from the homogenized tissue were extracted using the Folch procedure^33^. Heptadecanoyl (C17:0)-containing internal standards were added during the extraction. The extracts were evaporated using nitrogen, reconstituted in chloroform:methanol [2:1] and stored at −20 °C until further analysis. Cholesteryl esters, triglycerides, diglycerides, phospholipids and sphingomyelin were quantified using direct infusion/mass spectrometry according to previous publication^34^. Ceramides, dihydroceramides, glucosylceramides, lactosylceramides were quantified using ultra performance liquid chromatography tandem mass-spectrometry (UPLC-MS/MS)^35^.

### Echocardiography in mice

A baseline echocardiographic examination was performed in isofluorane-anesthetized mice using VisualSonics VEVO 770 system (VisualSonics), which includes an integrated rail system for consistent positioning of the ultrasound probe. The animals’ chests were shaved and hair removal gel was applied to minimize resistance to ultrasonic beam transmission. The mice were then placed on a heating pad and paws were connected to electrocardiographic (ECG) electrodes. A 45 MHz linear transducer (RMV 704) was used for imaging. An optimal parasternal long axis (LAX) cine loop (i.e. visualization of both the mitral and aortic valves, and maximum distance between the aortic valve and the cardiac apex) of >1000 frames/s was acquired using the ECG-gated kilohertz visualization technique. Parasternal short axis cine-loops were acquired at 1, 3, and 5 mm below the mitral annulus. End-diastolic and end-systolic LV volumes and ejection fraction were calculated by biplane Simpson’s formula using the 3 parasternal short-axis views and the parasternal long-axis view. M-mode measurements were performed (in the 3 mm level) using the leading-edge method. End-diastole was defined at the onset of the QRS complex, and end-systole was defined as the time of peak inward motion of the interventricular septum. At least three beats were averaged for each measurement. Myocardial velocities were measured for three consecutive cardiac cycles by pulsed-wave Doppler tissue imaging. The ultrasound beam was placed in parallel to wall motion in the short-axis view and three velocity signals during each cardiac cycle were measured: S, peak myocardial velocity during systole; E′, peak myocardial velocity during early diastole; A′, peak myocardial velocity during atrial contraction. Each echocardiographic examination was performed by an experienced echocardiographer and evaluation of the stored data was performed offline in a blinded fashion using VevoLab™ software system (VisualSonics).

### Dobutamine Stress Analysis

The mice were injected with dobutamine (2 µg/g body weight) intraperitoneally and echocardiography was performed as described above. Mice were excluded from the study if their heart rate did not increase by >100 beats/min. Therefore, one *Plin2*^−/−^ mouse was excluded that did not respond to doubutamine stress.

### Induction of Myocardial Infarction

To keep the mice sedated and support breathing during the operation, the mice were anesthetized with isoflurane, orally intubated and connected to a respirator (SAR-830 small animal ventilator, GENEQ) distributing a mixture of oxygen, air and 2–3% isoflurane. Electrodes were placed on the extremities and connected to an ECG-monitor to observe the cardiac rhythm during surgery. An incision was made between the 4^th^ and 5^th^ ribs, revealing the upper part of the anterior LV wall and the lower part of the left atrium. An extensive myocardial infarction was induced by ligation of the left anterior descending (LAD) coronary artery immediately after the bifurcation of the left coronary artery. The efficacy of the procedure was immediately verified by characteristic ECG-changes, and akinesia of the LV anterior wall. After verification of myocardial infarction, the lungs were hyperinflated, positive end-expiratory pressure was applied and the chest was closed. The mice received an intraperitoneal injection of 0.1 ml Temgesic to relieve postoperative pain and the mice recovered spontaneously when administration of the isoflurane was stopped.

### Mitochondrial respiration

Real-time measurements of oxygen consumption rate (OCR) was performed on a XFe96 Seahorse extracellular flux analyser (Seahorse Biosciences). The optimal seeding density and test compound concentrations were empirically determined prior to initiation of experiments. 5000 cardiomyocytes isolated from *Plin2*^+/+^ and *Plin2*^−/−^ mice were seeded in 80 µl of plating medium (MEM w Hanks BSS, 10% Calf Serum, 500 mM BDM, 100 U/ml Pencillin, 2 mM L-glutamine) in a XF96 cell culture plate (101085-004) (Agilent Technologies) and placed in 37 °C, 18% O_2_, 2% CO_2_ and after 2 hours the plating medium was replaced by short-term medium (MEM w Hanks BSS, 10% Fat-free BSA, Pencilin and L-glutamine). A mitostress assay was performed the following day. The short-term medium was changed to 180 µl pre-warmed assay medium (XF base medium supplied with 10 mM Glucose, 1 mM Sodium Pyruvate, 2 mM L-glutaime) (Agilent Technologies). The plate with the cardiomyocytes was incubated in 37 °C, 0% CO_2_ for 1 hour to pre-equiblirate. The mitostress test kit (103015-100; Agilent Technologies) was prepered by adding pre-warmed Oligomyocin (Oligo), carbonyl cyanide p-trifluoromethoxyphenylhydrazone (FCCP) and rotenone and antimycin A (R/A) to the injector ports A, B, C on a sensor cartridge (101085-004; Agilent Technologies). The final well concentration after optimization was as followed: 1 µM Oligo, 0,25 µM FCCP and 2 µM R/A. The cartridge was calibriated in the XF96 analyser (Seahorse Biosciences) after optimization. As previously shown, primary cardiomyocytes were not responsive to Oligomyocin^36^. OCR was detected under basal conditions, followed by sequential addition of Oligo, FCCP, R/A. This allowed for the calculation of basal and maximal respiration using the XF mito stress test report generator. All respiration rates were normalized to protein concentration in the respective well.

### Fatty Acid Oxidation

Heart ventricles were harvested from mice fasted for 4 hours, immediately minced, and homogenized in 20 volumes of ice-cold buffer (100 mM KCl, 40 mM Tris-HCl, 10 mM Tris base, 5 mM MgCl_2_·6H_2_O, 1 mM EDTA, and 1 mM ATP [pH 7.4]) with 10 up-and-down strokes of a motor-driven Teflon pestle and glass mortar. Homogenates (40 µl) were incubated with assay buffer (160 µl) containing 100 mM sucrose, 10 mM Tris-HCl, 10 mM K_2_HPO_4_, 100 mM KCl, 1 mM MgCl_2_·6H_2_O, 1 mM L-carnitine, 0.1 mM malate, 2 mM ATP, 0.05 mM coenzyme A, and 1 mM dithiothreitol (pH 7.4) supplemented with 8.6 µM [1-^14^C] oleic acid (0.1 μCi/reaction) (GE Healthcare) and 100 μM oleic acid complexed to bovine serum albumin (0.3%). The reaction was incubated for 60 minutes at 30 °C and terminated by transferral to a new Eppendorf tube containing 70% perchloric acid (100 µl). The ^14^C-labeled CO_2_ was trapped on a filter paper soaked in 1 M NaOH and placed in the lid of the tube. After a 60-minute incubation, the filter paper was removed, and the reaction mixture was centrifuged at 14 000 rpm for 10 minutes to obtain the acid-soluble metabolites (ASM) from the supernatant. Radioactivity of ASM and CO_2_ was determined with an LS6500 scintillation counter (Beckman Coulter). Fatty acid oxidation was quantified as [(dpm − BL)/SA]/[mg protein × time (in hours) of reaction mixture incubation]), where dpm is disintegrations per minute, BL is the dpm of blank wells, and SA is the fatty acid–specific radioactivity. Total fatty acid oxidation was calculated as the sum of ASM and CO_2_.

### Immunofluorescent stainings on primary cardiomyocytes

Primary cardiomyocytes were washed with PBS and then fixed with 4% formaldehyde for 5 minutes. The cells were washed again with PBS and blocked with 1% BSA in PBS for 20 minutes containing 0,1% saponin. After blocking, the cells were incubated with the following primary antibodies for Plin2 (B3121; LSBio), Plin3 (NB110-40764; LSBio), Plin5 (GP31; Progen), ATP5s (ab14748; Abcam) and Lamp-1 (ab25245; Abcam) with 1% BSA in PBS together with 0, 1% saponin for 2 hours. Corresponding secondary antibodies were added together with 1% BSA, 0, 1% saponin in PBS, for 1 hour after a washing step. The cells were thereafter incubated in 0.3% Oil Red O (ORO) solution for 20 minutes, and then washed properly with dH_2_O. Nuclei were stained with DAPI (2 µg/ml) (Thermofisher Scientific) that was added with dH_2_O for 5 minutes before mounting. The cells were mounted on glass slides with Prolong Gold Antifade Mounting medium (ThermoFisher Scientific).

### Imaging

The immunohistochemistry slides were scanned with a Lecia TCS SP5 confocal microscope (Leica Microsystems). LAS X was used as image software (Leica Microsystems). Representative images are shown in all figures and cardiomyocytes were isolated from a minimum of 4–5 mice per genotype, minimum 4 slides per mouse and 4 cells per slide.

### Analysis of lipid droplets and ATP5s

Visiopharm image analysis software (Visiopharm, Denmark) was used for image analysis and quantification of either ORO or ATP5s positive area (pixels).

### Analysis of length and thickness of cardiomyocytes

For analysis of cardiomyocyte length and thickness, LAS X was used. The length and thickness was measured with the ruler that is included in the programed.

### Analysis of colocalization

For colocalization analysis, images were segmented into three compartments; whole-cell, membrane and intracellular, and were quantified with regards to mean signal intensity for ORO and LAMP1 in ImageJ 1.52d, according to Schneider *et al*.^37^ with the use of an in-house macro. Mander’s overlap coefficients were calculated using the ImageJ plugin Coloc 2 (v3.0.3, https://imagej.net/Coloc_2) with Costes’ automatic threshold^38^ for the membrane and intracellular compartments.

### Autophagy flux in primary cardiomyocytes

Isolated cardiomyocytes were starved for 2 hours with Earle’s Balanced salt solution (EBSS) (Thermofisher Scientific) with or without 25 µM Chloroquine diphosphate salt (SIGMA) before harvest.

### Statistical analysis

Values are expressed as mean ± SEM. Differences between groups were analysed with unpaired two-tailed *t* tests using GraphPad Prism Software. *p* < 0.05 was considered statistically significant.

## Supplementary information

Supplementary information
