# Supplementary material for: Plin2-deficiency reduces lipophagy and results in increased lipid accumulation in the heart
Source: Sci Rep. 2019 May 6;9:6909. doi: 10.1038/s41598-019-43335-y (PMC6502866; doi:10.1038/s41598-019-43335-y)
Supplement: Supplementary file 1 — Supplementary information [file 41598_2019_43335_MOESM1_ESM.pdf]

SUPPLEMENTARY INFORMATION

Plin2-deficiency reduces lipophagy and results in increased lipid accumulation in the heart

Ismena Mardani[1], Knut Tomas Dalen[2], Christina Drevinge[1], Azra Miljanovic[1], Marcus Ståhlman[1], Martina Klevstig[1], Margareta Scharin Täng[1], Per Fogelstrand[1], Max Levin[1], Matias Ekstrand[1], Syam Nair [3], Björn Redfors[1], Elmir Omerovic[1], Linda Andersson[1], Alan R. Kimmel[4], Jan Borén[1] and Malin C. Levin[1#]

[1]Department of Molecular and Clinical Medicine / Wallenberg Laboratory, Institute of Medicine, the Sahlgrenska Academy at University of Gothenburg and Sahlgrenska University Hospital. [2]Department of Nutrition, Institute of Basic Medical Sciences, University of Oslo, Oslo, Norway. [3] Centre of Perinatal Medicine and Health, Institute of Neuroscience and Physiology, the Sahlgrenska Academy at University of Gothenburg, Sweden. [4]Laboratory of Cellular and Developmental Biology, National Institute of Diabetes and Digestive and Kidney Diseases, National Institutes of Health, Bethesda, MD, USA.

[#]Correspondence to:
Malin Levin, Wallenberg Laboratory, Sahlgrenska University Hospital
413 45 Gothenburg, SWEDEN, malin.levin@wlab.gu.se, phone no: +46-31-342 4153.

Figure S1

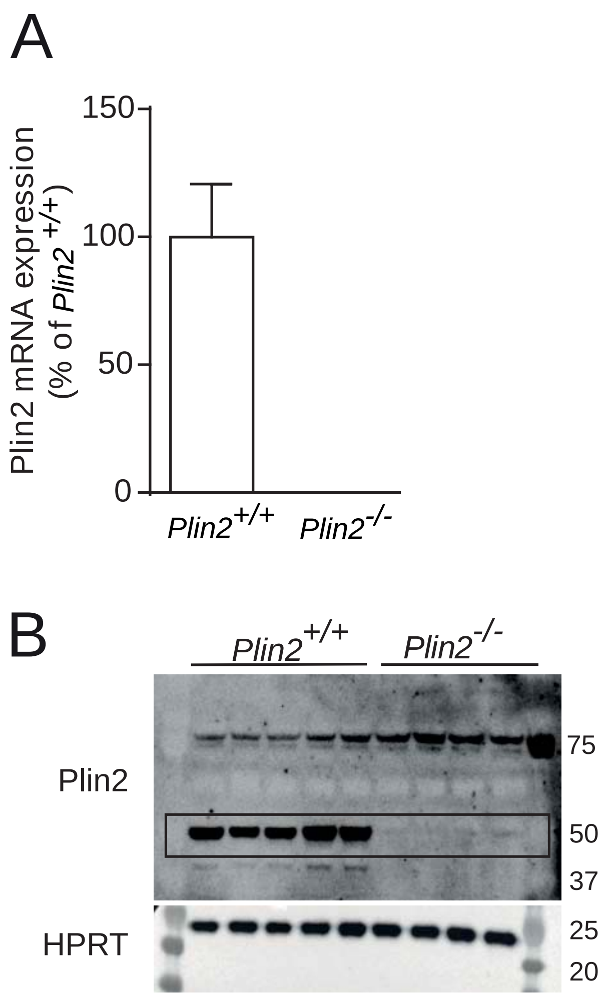

Figure S1. Characterization of *Plin2*$^{-/-}$ mice. (A) Quantitative real-time PCR of Plin2 mRNA in hearts of *Plin2*$^{+/+}$ and *Plin2*$^{-/-}$ mice (n=5). (B) Immunoblot analysis of Plin2 protein lysates from hearts of *Plin2*$^{+/+}$ and *Plin2*$^{-/-}$ mice, 4 hours after fasting (n=4-5). Data presented as mean±SEM.

Figure S2

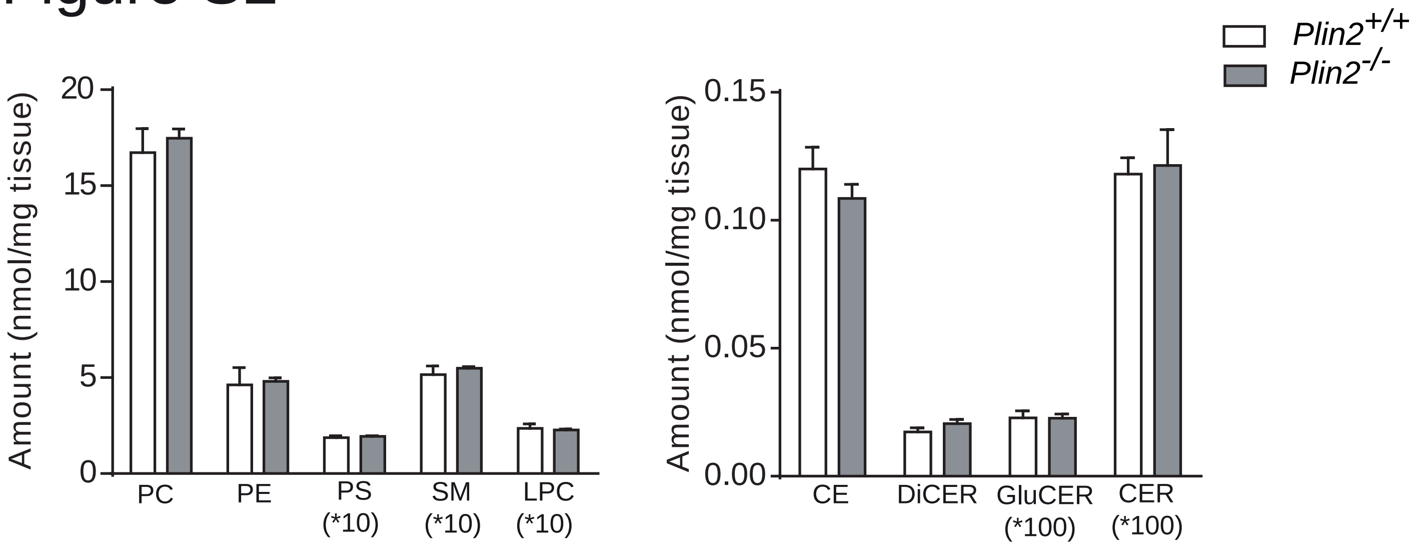

Figure S2. Lipid content in hearts of Plin2$^{-/-}$ and Plin2$^{+/+}$ mice. After a 4 hours fast (n=6-7), data presented as mean±SEM. PC, phosphatidylcholine; PE, phosphatidylethanolamine; PS, phosphatidylserine; SM, sphingomyelin; LPC, lysophosphatidylcholine; CE, cholesterol esters; CER, ceramide; DiCER, dihydroceramide; GluCER, glycosylceramide.

Figure S3

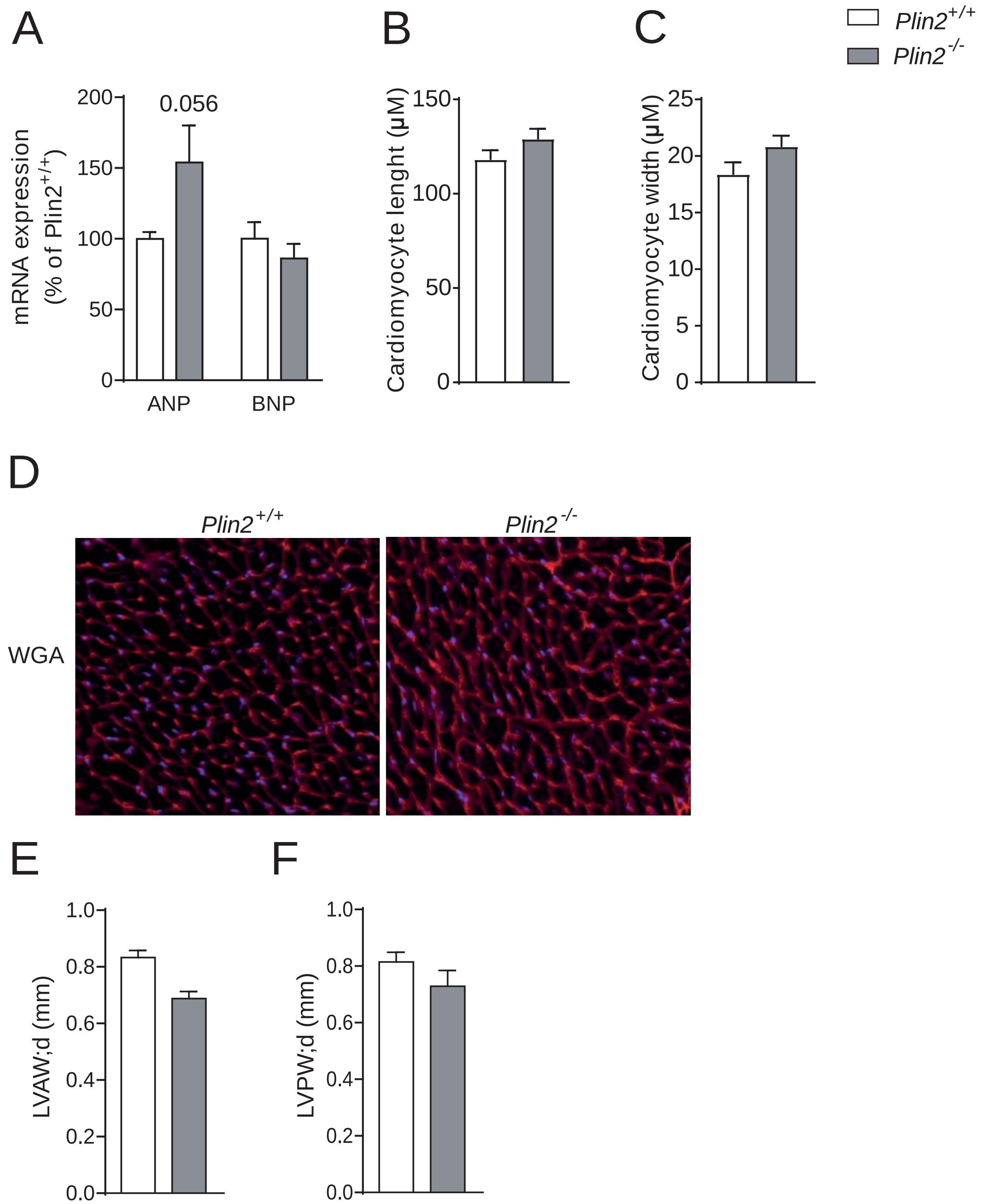

Figure S3. Natriuretic peptide expression and heart dimensions in Plin2$^{+/+}$ and Plin2$^{-/-}$ mice.
(A) Quantitative real-time PCR of Plin2 mRNA in hearts of *Plin2*$^{+/+}$ and *Plin2*$^{-/-}$ mice (n=5).
(B-C) Cardiomyocyte length and width (µM) (n=4-5). (D) Representative WGA staining of heart sections from *Plin2*$^{+/+}$ and *Plin2*$^{-/-}$ mice. (E) Left ventricle anterior wall thickness in diastole (LVAW;d) and (F) left ventricle posterior wall thickness in diastole (LVPW;d) in Plin2+/+ and Plin2-/- mice (n=3). Data presented as mean±SEM.

Figure S4

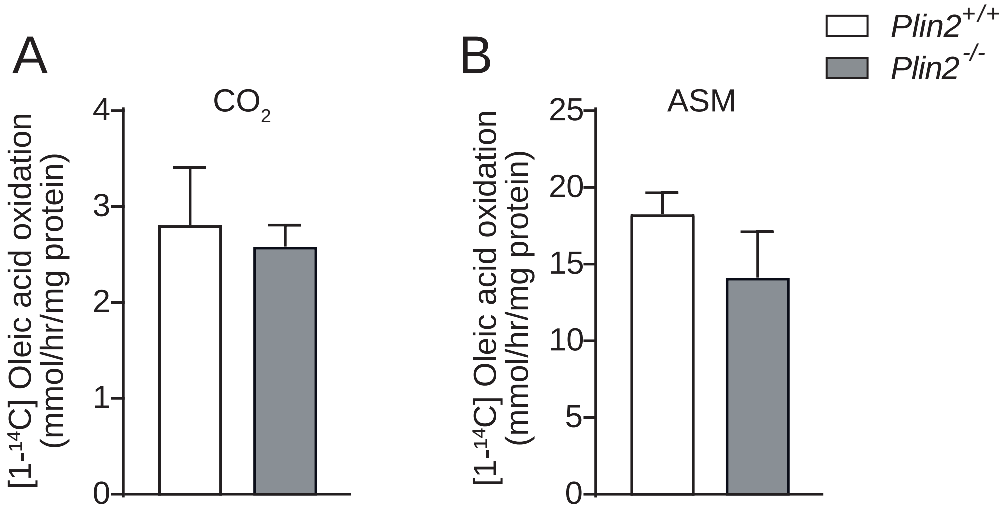

Figure S4. Fatty acid oxidation in Plin2$^{+/+}$ and Plin2$^{-/-}$ hearts. Produced (A) CO_2 and (B) ASM (n=3). Data presented as mean±SEM.

Figure S5

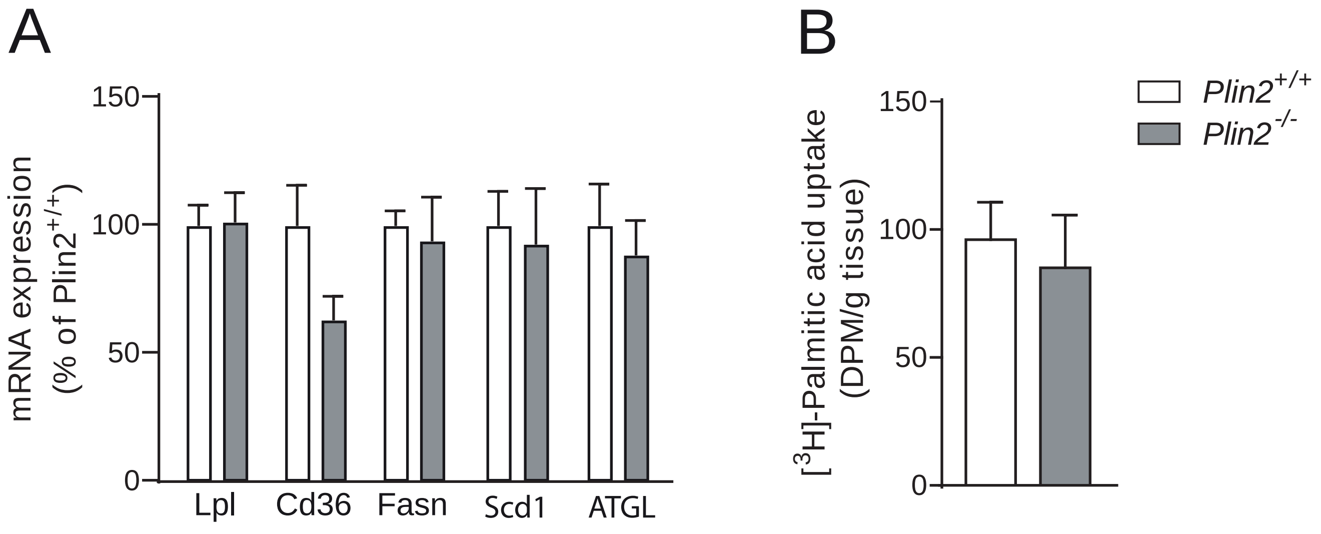

Figure S5. Markers of lipolysis and lipogenesis and lipid uptake in *Plin2*$^{+/+}$ and *Plin2*$^{–/–}$ hearts. (A) mRNA expression of Lpl, Cd36, Fasn, Scd1 and ATGL (n=4). (B) [3H]-Palmitic acid uptake in *Plin2*$^{+/+}$ and *Plin2*$^{–/–}$ hearts (n=2-4). Data presented as mean±SEM.

Figure S6

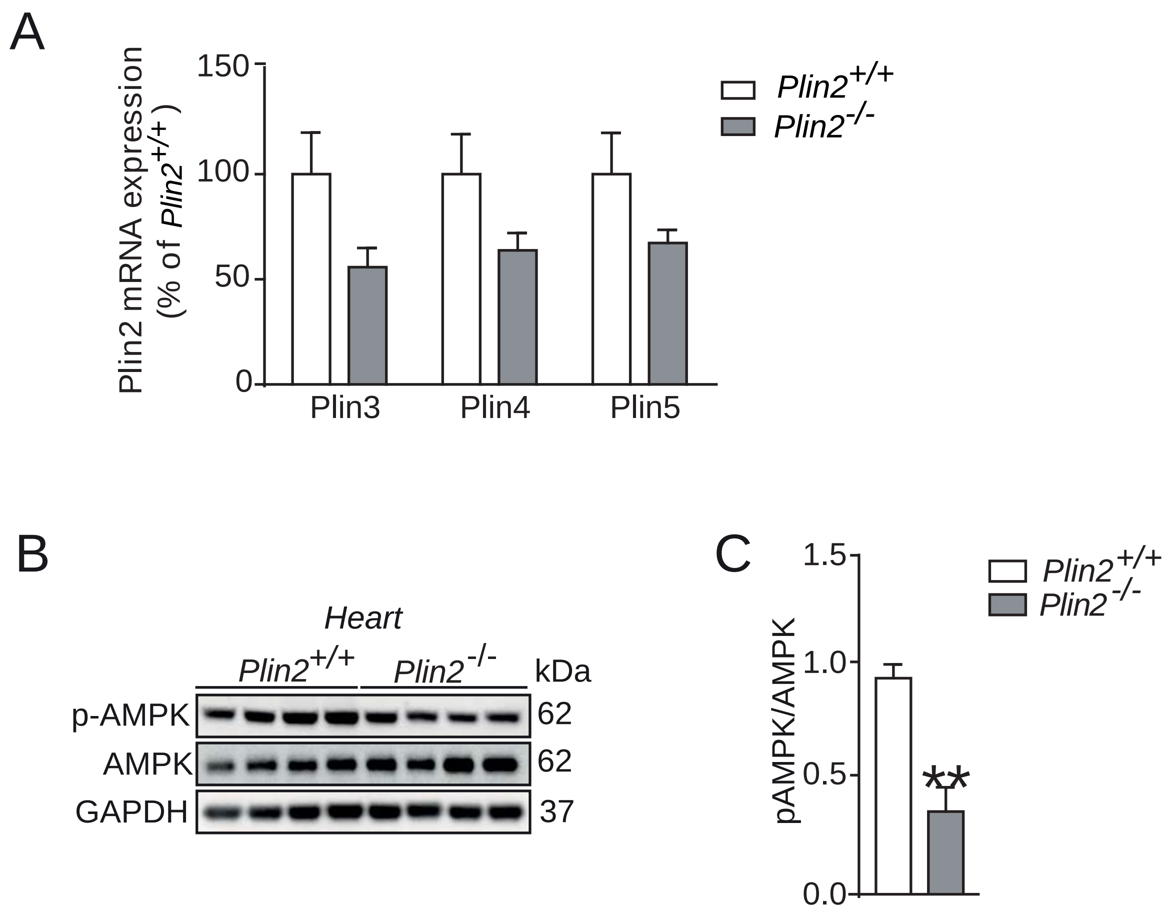

Figure S6. A) mRNA expression of Plin3, Plin4 and Plin5 in hearts of *Plin2*$^{+/+}$ and *Plin2*$^{–/–}$ mice. After a 4 hours fast (n=4-5). (B) Immunblot analysis of p-AMPK and AMPK in lysates from *Plin2*$^{+/+}$ and *Plin2*$^{–/–}$ hearts after O/N fasting. Full-length blots are presented in Supplementary Figure S10. (C) Quantification of immunoblot analysis in C (n=4). Data presented as mean±SEM.

Figure S7

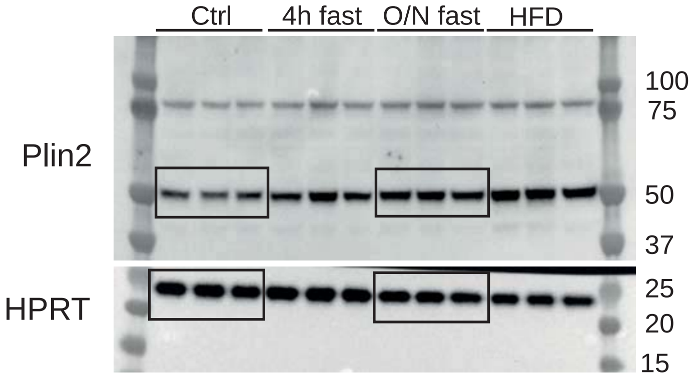

Figure S7. Full western blots of Figure 1A used for quantification.

Figure S8

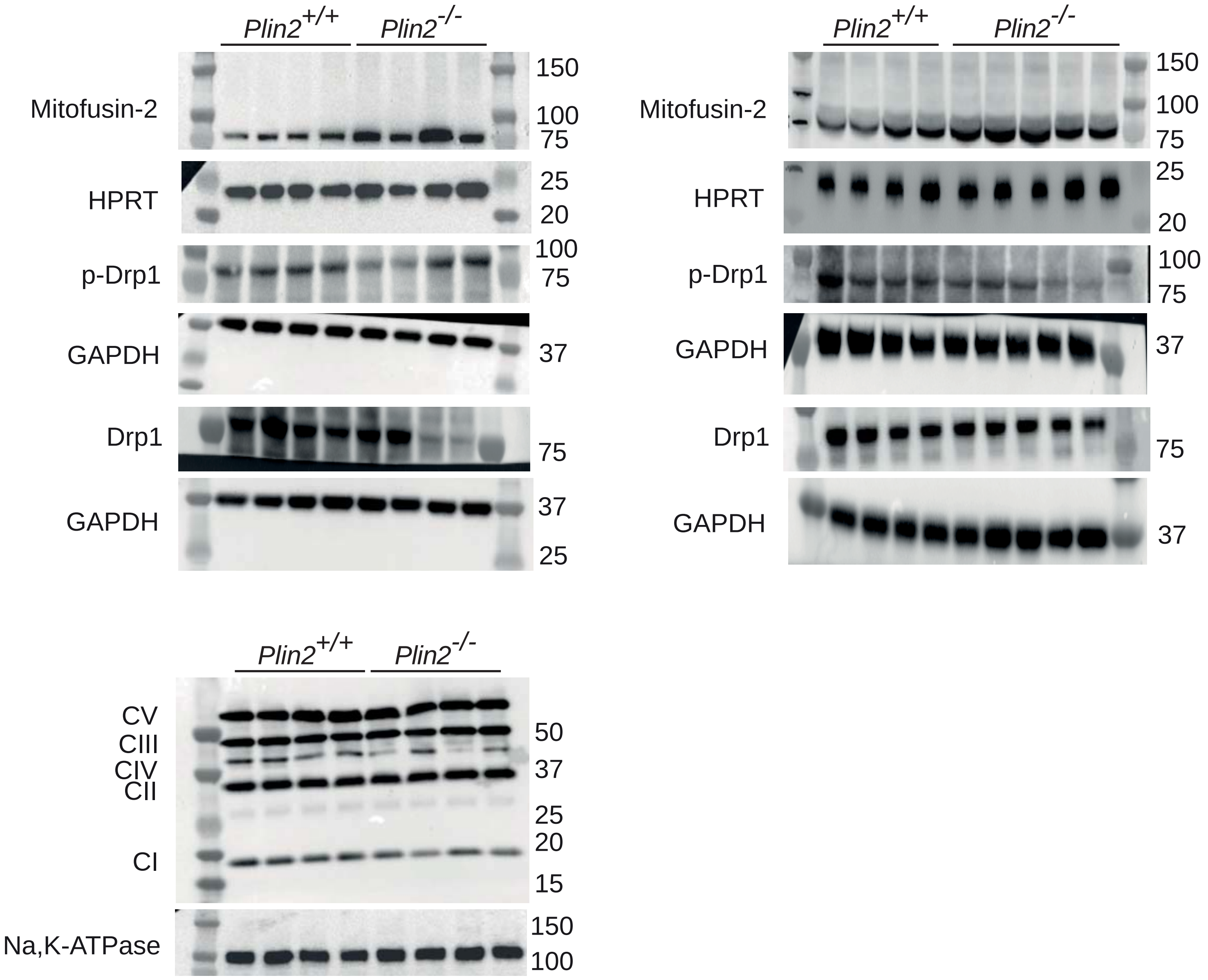

Figure S8. Full western blots of Figure 4D and F used for quantification.

Figure S9

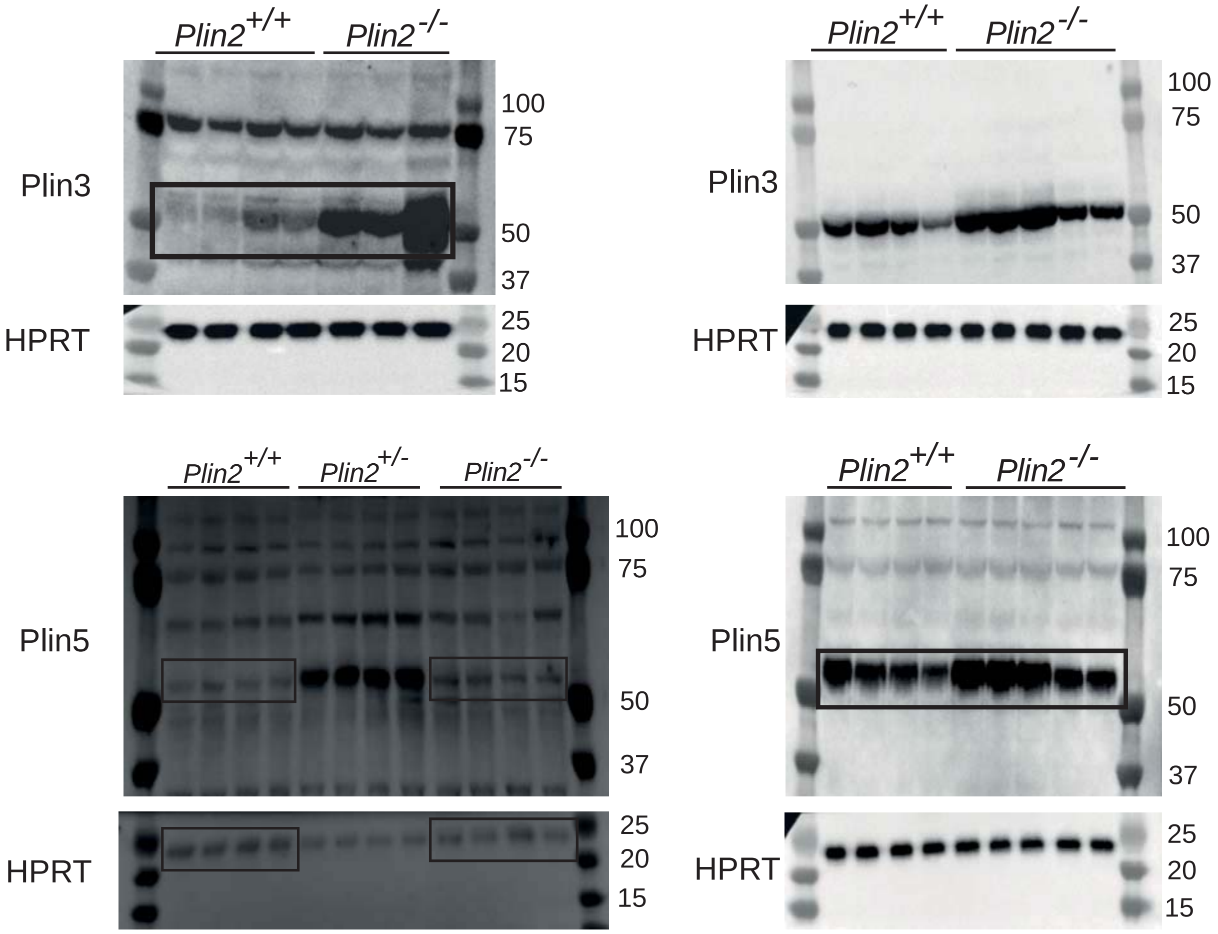

Figure S9. Full western blots of Figure 5A used for quantification.

Figure S10

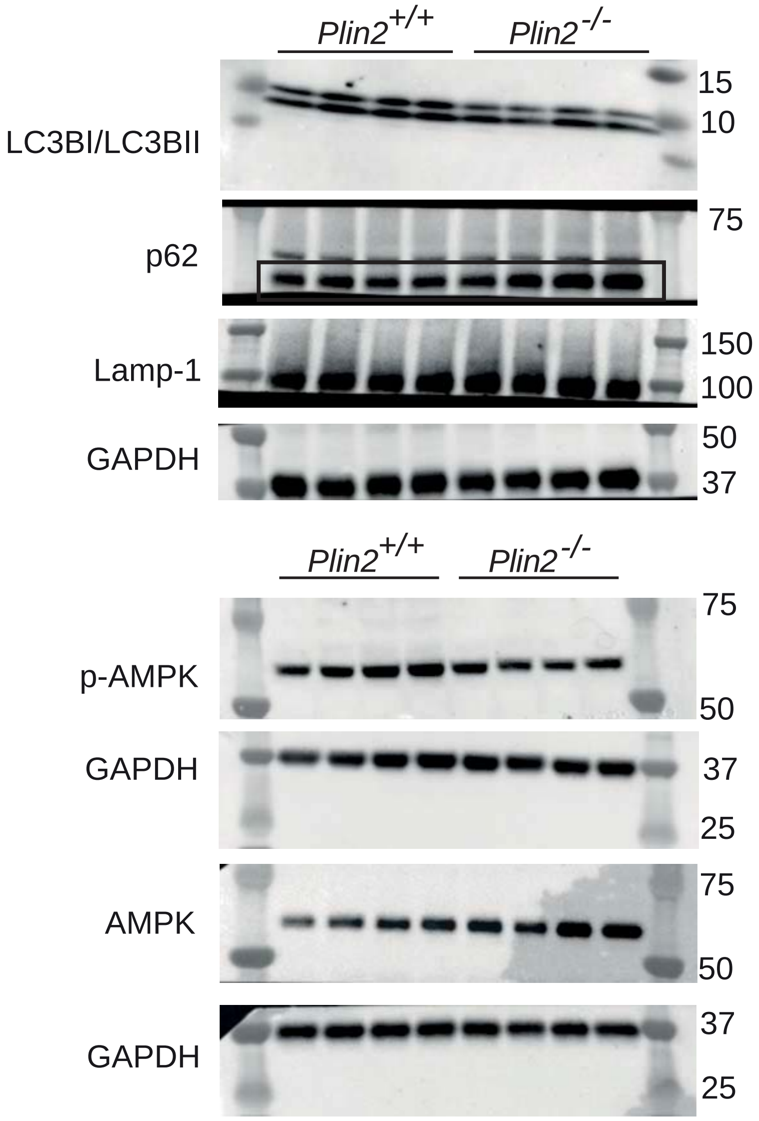

Figure S10. Full western blots of Figure 6A and F and Figure S6B used for quantification.

Figure S11

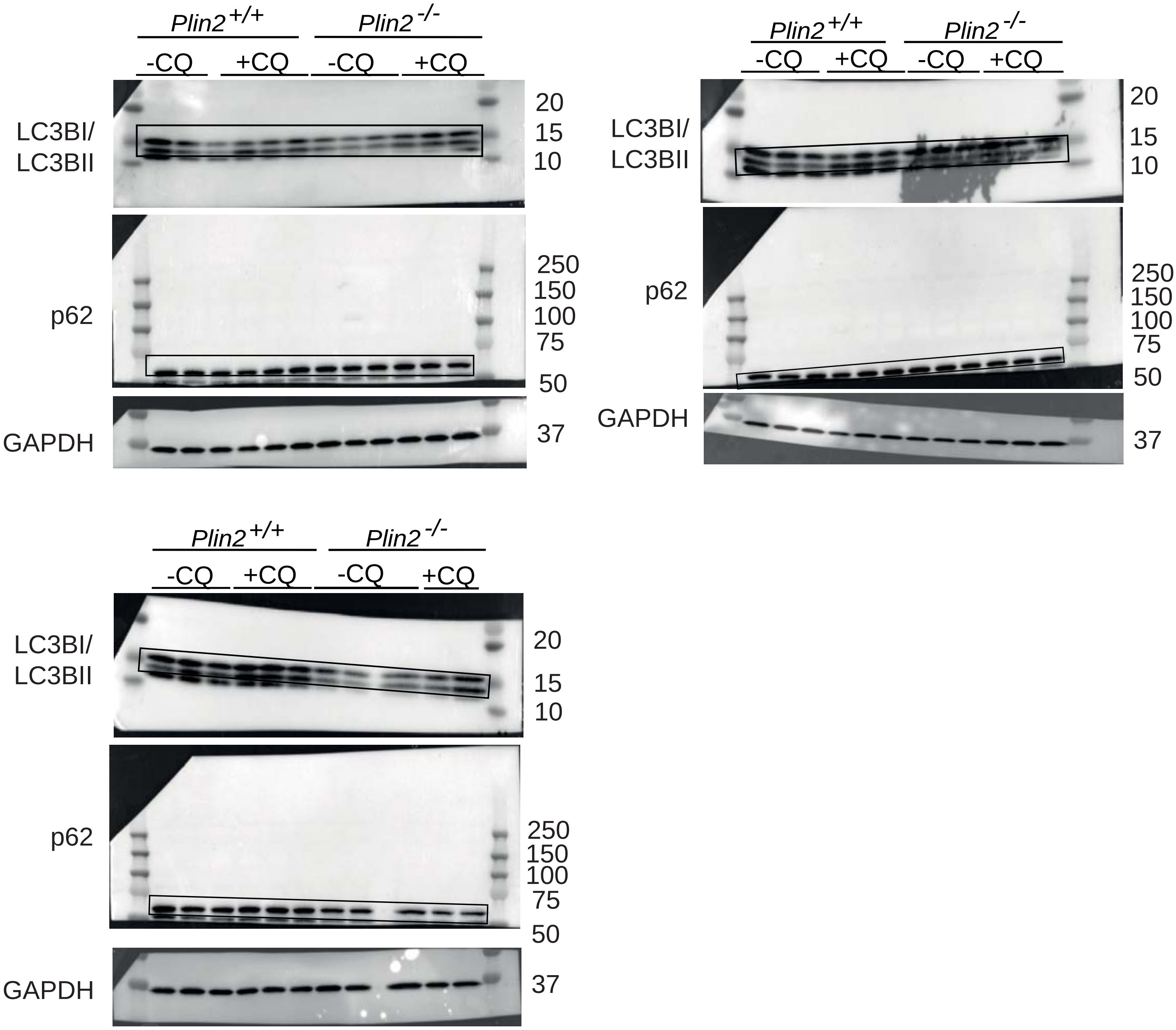

Figure S11. Full western blots of Figure 6C used for quantification.
